# Deficient or Normal Growth Hormone Secretion in Polish Children with Short Stature: Searching for Clinical Differences

**DOI:** 10.3390/biomedicines12081673

**Published:** 2024-07-26

**Authors:** Katarzyna Anna Majewska, Magdalena Tchorzewska-Skrobich, Paulina Wais, Dominik Majewski, Monika Naskręcka, Andrzej Kędzia

**Affiliations:** 1 Department of Pediatric Diabetes, Auxology and Obesity, Poznan University of Medical Sciences, 60-572 Poznan, Polandakedzia@ump.edu.pl (A.K.); 2 Department of Internal Medicine, Poznan University of Medical Sciences, 60-780 Poznan, Poland; dominik.majewski@ump.edu.pl; 3 Department of Applied Mathematics, Poznan University of Economics and Business, 61-875 Poznan, Poland

**Keywords:** short stature, growth hormone, growth hormone deficiency, children

## Abstract

Short stature affects approximately 2.5% of children. Some of them, when diagnosed with growth hormone deficiency (GHD), benefit from recombinant human growth hormone (rhGH) therapy; in others, this treatment is controversial. We aimed to present the clinical characteristics of Polish short stature children in the context of current GHD diagnostic standards, as obtaining more data gives a broader foundation for the potential modifications of diagnostic and therapeutic recommendations. This retrospective analysis was based on a cohort of 277 short stature children divided into two subgroups depending on their peak growth hormone (GH) cutoff level, set at 10 ng/mL: 138 had growth hormone deficiency (GHD) and 137 had normal growth hormone secretion (GHN). These subgroups were then compared based on the extracted clinical data. In the obtained result, no significant differences between the GHD and GHN subgroups were found in any of the variables, including the following: gender distribution, birth weight, bone age delay, height SDS, IGF-1 SDS, vitamin D levels, celiac disease indices, prevalence of hypothyroidism or anemia. As our results point to major clinical similarities between the GHD and GHN children, it seems that distinguishing patients with normal GH secretion from those with deficient GH secretion based on a 10 ng/mL cutoff value might not be clinically relevant.

## 1. Introduction

The diagnosis of short stature is based on statistical criteria. The population normal values of body height cover a range of two standard deviations (SD) from the average matched for age and gender. Therefore, a child’s height below −2SD is considered short stature [1,2]. Thus defined, it affects approximately 2.5% of all children, which makes it a substantial pediatric problem. 

Many factors (genetic, hormonal, and environmental) are involved in the regulation of human growth, and abnormalities in these factors may significantly disturb this process, leading to short stature. Numerous specific clinical conditions are associated with growth deficits—growth hormone deficiency (GHD), thyroid dysfunction, intestinal malabsorption, anemia, kidney failure, congenital heart defects, certain genetic syndromes, and others [2,3,4,5,6]. Some of these diseases are related to distinct, clear signs, and typical patient history; others may present atypical or scant symptoms. Due to the risk of various pathologies related to growth disorders, every short child should be subject to pediatric care with growth velocity evaluation and appropriate laboratory tests performed as required [7]. However, many of these children present no signs and symptoms of any other disease and meet the criteria of idiopathic short stature, familial short stature or simply demonstrate a constitutionally delayed process of growth and puberty [1,8]. Distinguishing healthy short children from those who need specific treatment requires careful clinical observation. Early and proper diagnosis with the implementation of appropriate treatment is essential for growth improvement and for preventing the consolidation of a problem in adulthood, as a therapeutic intervention is effective only in the developmental period, before the termination of growth. 

A particular group of children with short stature are those that suffer from GHD. These patients require long-term therapy with recombinant human growth hormone (rhGH), and its early implementation is crucial for satisfactory growth improvement [9,10]. The suspicion of this disease requires the referral of the child to a specialist center, and in order to set the diagnosis, specific stimulation tests are needed [1,11,12]. GHD is theoretically associated with certain features, such as significant growth restriction accompanied by delayed bone age and reduced insulin-like growth hormone 1 (IGF-1) levels [1,7]. But how do these features correspond with the results of growth hormone (GH) stimulation tests? And what exactly causes the difference?

The aim of this study was to present the basic clinical characteristics of Polish children with short stature, performed in the context of GH secretion, and evaluated according to current local guidelines. Obtaining more data would give a broader foundation for a constructive discussion regarding existing diagnostic and therapeutic recommendations and their potential modifications.

## 2. Materials and Methods

The study was based on a retrospective analysis of medical data collected between 2014 and 2022. The cohort covered 277 children with short stature from the region of central and western Poland, aged from 2 to 17 years (mean 8.89 ± 3.38); these were all patients of The Karol Jonscher Teaching Hospital of the Poznan University of Medical Sciences, admitted due to diagnostic procedures establishing causes of growth failure. Inclusion criteria covered a height SDS (hSDS) value below −2 and the completion of diagnostic procedures. Exclusion criteria covered those previously diagnosed with accompanying chronic diseases, e.g., multiple pituitary hormone deficiency, malabsorption, genetic syndromes, kidney failure, and others. All patients underwent a physical examination, with the exact measurements of height and an assessment of general health. Height measurements were performed using a stadiometer with an accuracy of 1 mm. Based on the collected data, for all the children, an appropriate hSDS (standard deviation score of child’s height in relation to gender and age) was calculated in accordance with the guidelines [13,14,15]. 

For all included children, the following laboratory tests results were extracted: thyrotropin (TSH), thyroxine (FT4), anti-tissue transglutaminase and anti-gliadin antibodies, 25-OH vitamin D, insulin-like growth factor 1 (IGF-1), and also the blood count (hemoglobin, HGB). Celiac disease diagnosis was based on positive TTG IgA, confirmed via the results of biopsy. Hypothyroidism was classified as an elevated TSH level (above 5 µIU/mL; subclinical hypothyroidism when TSH between 5 and 10 µIU/mL). Vitamin D deficiency was defined as a serum level below 20 ng/mL, while vitamin D insufficiency was defined as a serum level between 20 and 30 ng/mL. Among children with vitamin D levels lower than 20 ng/mL, additional analysis was performed, considering ranges 15–20 and below 15 ng/mL. Anemia was classified based on the following laboratory standards for HGB levels, adjusted to be appropriate for age and gender: in boys and girls aged 2–6 years, below 10.9 g/dL; 7–12 years, below 12.0 g/dL; girls older than 12 years, below 12.0; boys older than 12 years, below 14.0 g/dL.

According to the guidelines of the Polish Coordination Team for the Use of Growth Hormone, the patients had sleep tests performed with blood samples taken before and 30, 60, 90, and 120 min after falling asleep; they also underwent 2 stimulation tests with glucagon (0.03 mg/kg of body weight) and insulin (0.1 units/kg of body weight), or clonidine (100 mcg/m^2^). For the purpose of this study, the children were divided into 2 subgroups depending on GH test results; those who had a peak GH level below 10 ng/mL were included in the GHD subgroup, while those who presented a peak GH level above 10 ng/mL qualified as normal GH secretion (GHN) and were included in the GHN subgroup. These subgroups were then compared on the remaining clinical data collected. 

Due to IGF-1 normal range dependence on age and gender, for the purpose of further analysis, IGF-1 SDS values were calculated following the appropriate standards. Calculations were performed for every patient according to Bidlingmaier et al. [16], with the formula z = {[(IGF1/M)^L] − 1}/L × S, where the LMS values were extracted from LMS charts appropriate for age and gender [16].

Bone age assessment was based on the X-ray of the wrist and hand, according to the Greulich and Pyle method. Bone age delay was determined as the difference between the bone and the calendar age. Also, the bone age/calendar age (BA/CA) index was calculated. 

Birth weight data were extracted from the patient’s history, and the definition as small for gestational age (SGA) was determined on the basis of appropriate population reference ranges [17]. 

The IBM SPSS Statistics 26 program was used for the statistical analysis. Throughout the study, the *p* < 0.05 threshold was adopted as the significance level. Examined variables were presented as mean values and standard deviations. The Shapiro–Wilk test was used to test the normality of distributions. Due to the lack of a normal distribution among the data, in further analysis, a non-parametric Mann–Whitney U test was selected to compare the results. In order to compare structure indicators, the z-test was used. 

The study was conducted following the guidelines of the Declaration of Helsinki and was based on the approval of the local Ethics Committee at the Poznan University of Medical Sciences (No 318/16, 1141/19).

## 3. Results

The mean values and standard deviations of the examined variables are presented in Table 1. It shows the results for all short stature children of the study group, as well as their results following their division into subgroups; these subgroups were defined as patients with a peak GH level <10 ng/mL classified as growth hormone deficiency (GHD), and those with a peak GH level ≥10 ng/mL and normal growth hormone secretion (GHN). There was a clear predominance of boys over girls, but it presented similarly in all the analyzed groups, with no significant difference between the GHD and GHN subgroups (*p* = 0.582). A subsequent analysis with the use of the Mann–Whitney test revealed no significant differences between these subgroups in other variables: bone age delay (*p* = 0.682), BA/CA (*p* = 0.122), hSDS (*p* = 0.517), IGF-1 (*p* = 0.078), IGF-1 SDS (*p* = 0.319), TSH (*p* = 0.126), FT4 (*p* = 0.570), 25-OH vitamin D (*p* = 0.873), HGB (*p* = 0.200), and birth weight (*p* = 0.273). There were also no significant differences found with the use of the z-test in the percentage occurrence of the following conditions: celiac disease (*p* = 0.749), hypothyroidism (*p* = 0.646), anemia (*p* = 0.147), vitamin D deficiency (*p* = 0.435), vitamin D insufficiency (*p* = 0.052), regular vitamin D intake (*p* = 0.270), and birth weight small for gestational age (SGA) (*p* = 0.418)—the detailed results are presented in Table 2.

Regarding vitamin D evaluations, an additional analysis was performed in patients with its serum concentrations below 20 ng/mL. In the obtained results, we found a vitamin D level between 15 and 20 ng/mL in 20.6% of all short-statured children (21.9 and 19.4% in GHD vs. GHN children, respectively, *p* = 0.608 in z-test). Only 15.5% of all short-statured children had a vitamin D serum concentration below 15 ng/mL (13.9 vs. 17.3% in GHD and GHN children, respectively, *p* = 0.436 in z-test).

## 4. Discussion

GH is the key hormone involved in the regulation of human body growth. It is secreted by the pituitary gland in a pulsatile manner, mainly during sleep at night. Its efficient synthesis and release are crucial for normal growth in children [18]. Isolated GHD may be caused by genetic factors or structural changes in the area of the hypothalamus and pituitary, but the most commonly diagnosed form is idiopathic, with the cause of disease remaining unknown [2,6,18,19]. The incidence of its congenital form is estimated at the level of 1 in 4000 to 1 in 10,000 live births, with the familial occurrence from 3 to 30% depending on the population studied [1,6,20]. 

In most countries, GHD diagnosis in short stature children requires two different GH stimulation tests. A failure to respond to both tests is needed to diagnose GHD, and a sufficient GH level in at least one excludes the diagnosis [1,11,12]. GH cutoffs have gradually increased to 10 ng/mL; however, the diagnostic criteria vary from center to center (mainly between 6.7 and 10 ng/mL) [11]. Generally, GH peak levels below 5 ng/mL point at complete or severe GHD, and levels between 5 and 10 ng/mL point towards partial or moderate GHD [21,22]. However, other authors suggest the diagnosis of the severe form when the peak GH concentration is below 3 ng/mL [1,12].

In Poland, the diagnosis of GHD requires performing two pharmacological stimulation tests with a cutoff set at 10 ng/mL, and additionally, up to 2022, all children must have also had a sleep test—exhibiting a short profile of spontaneous night GH secretion [11,19]. rhGH treatment, based on the national therapeutic program, is currently available for children fulfilling the criteria of GHD or SGA, but not for those diagnosed with idiopathic short stature. 

For the purposes of this study, the basic aspects of diagnostic evaluation of children with short stature were selected: gender, birth weight, bone age delay, the severity of height deficiency, IGF-1 serum concentrations, thyroid function parameters, vitamin D status, occurrence of anemia, and indices of celiac disease. All of this was in the context of GH secretion assessment. 

Our first observations point to differences in gender distribution, with more boys than girls diagnosed with growth hormone deficiency. This seems to be consistent with other studies worldwide. The first reports indicating male predominance among patients with GHD treated with rhGH date back to 1990, and this predominance has persisted for three decades, which is confirmed by numerous studies [20,23,24]. This observation, however, should not be interpreted directly. As short stature is diagnosed in both girls and boys in the same way—when their height is more than 2 SD below the mean for their appropriate age and gender—comparable numbers of girls and boys fulfill the definition of short stature. Consequently, if there are more boys than girls diagnosed with GHD, this would suggest GHD to be more frequent in boys—but is it really? In our study, the prevalence in boys was similar in all the analyzed groups—not only in children with GHD, but also in those with normal GH secretion, as well as in all patients with short stature when analyzed together. Therefore, it seems that among short children, simply more boys than girls are referred for diagnostic procedures, so, consequently, proportionally more boys are also diagnosed with GHD. Our observation supports the thesis that girls are generally under investigated in terms of growth retardation; this is probably due to a worldwide acceptance for girls and women to be short, but not boys and men. However, according to current guidelines, they should be evaluated and treated appropriately, similarly to boys [1,20,24]. 

Another analyzed aspect was the birth weight, also in terms of SGA diagnosis. Children born small for gestational age have an increased risk of further growth failure [25]. The definition of SGA reflects a birth weight below 2 SD of the appropriate population. Most SGA children present catch-up growth in early childhood and reach normal height; however, approximately 10% of them remain short also during adolescence and adulthood [26,27]. Short children born as SGA may present either normal or deficient GH levels. In our study, we found no statistically significant differences in terms of birth weight and SGA occurrence between children with normal and deficient growth hormone secretion. 

Bone age is an objective indicator of biological maturation in children. It is typically delayed in patients with GHD, but delays are common also in other conditions such as hypothyroidism, undernutrition, SGA during the prepubertal stage, or a constitutional delay of growth [1,26]. On the other hand, in GHD children, bone age might not be markedly different from the calendar age in the case of some other coexisting diseases, e.g., obesity [1]. In our study, bone age delay, as well as the BA/CA index in children with the diagnosis of GHD, did not differ significantly from those with normal growth hormone secretion. What seems important is that the severity of growth restriction evaluated as hSDS also failed to show statistically significant differences. 

The IGF-l serum level is claimed to be substantial for GHD diagnosis and is frequently used as a screening parameter. However, IGF-1 evaluation carries a significant risk of inaccuracy [28]. Its production is regulated not only by pituitary function, as an important influencing factor is the nutritional state; IGF-1 is also sensitive to current food intake, and therefore blood sampling should be performed in the morning, while abstaining from food. If the patient has eaten prior to the testing, the result would be higher. Relatively higher values are also observed in obesity. On the other hand, undernutrition is related to lower IGF-1 serum concentrations. What is important is that nutrient deficiency should also be taken into account among short stature children in developed countries, as some of these children may present a strongly selective appetite, or may have undiagnosed intestinal malabsorption syndrome, causing a disturbed growth pattern [29]. As IGF-1 normal ranges change depending on age, gender, and stage of puberty, consequently their levels require SDS calculation. These calculations adjust obtained results directly to age and gender and, to some extent, indirectly also to the stage of puberty. It has been assessed that if IGF-1 SDS values are above 0, the diagnosis of GHD is unlikely [7,30,31]. In our study, the mean IGF-1 SDS was below 0 in both the GHD and GHN children. Even though the mean IGF-1 levels and IGF-1 SDS were slightly lower in GHD than in GHN children, the differences did not reach a statistical significance. It is worth noting that the above also corresponds to findings of other authors, suggesting that IGF-1 itself is not reliable in distinguishing children with normal and deficient growth hormone secretion [7,12,28], although the assessment of the diagnostic value of IGF-1 was not the subject of our study.

Celiac disease is one of the important causes of malabsorption in developed countries. It is a systemic disease associated with an inappropriate immune response to gluten, with a typical remodeling of the small intestinal mucosa and villous atrophy. It affects approximately 1% of the European population. Classical presentation used to cover primarily gastrointestinal symptoms, but currently extraintestinal symptoms, such as growth failure or iron-deficiency anemia, have become dominant [32,33]. Short stature seems to affect 10–40% of children at the time of celiac disease diagnosis and is the most common extra-intestinal symptom. The pathogenesis of growth failure in celiac disease covers malnutrition itself, but it seems to be also related to the dysregulation of GH/IGF-1 axis [29,33]. It has been suggested that up to 8% of children undergoing a diagnostic process due to short stature may be diagnosed with celiac disease [33]. In our study, indices of celiac disease were found in 4% of all short stature children, with no significant differences between the GHD and GHN group. 

Another important issue in the clinical evaluation of short stature children is anemia. Its prevalence in this group of patients is well documented, and mutual relations are multidirectional. Infants and young children with anemia generally present growth delay. It has been observed that congenital forms of anemias, such as Fanconi or Blackfan–Diamond, are often related to short stature [34,35]. Iron deficiency anemia may be one of the symptoms of celiac disease, also related to impaired growth or undernutrition resulting in a failure to thrive [7,29,36]. However, on the other hand, the GH/IGF-1 axis is involved in the process of hematopoiesis. Anemia seems to occur more frequently in children with GHD, who present generally lower hemoglobin and red blood cells than healthy children. What is important is that growth hormone therapy causes a significant improvement in erythropoiesis indices in patients with GHD [36]. Our analysis has shown a similar occurrence of anemia in children with deficient and normal growth hormone secretion. 

Acquired juvenile hypothyroidism is rarely diagnosed as a cause of short stature in children. Other typical symptoms, like drowsiness, reduced activity, or difficulty in concentration, occur earlier and, therefore, the diagnosis of hypothyroidism with the implementation of appropriate treatment should be made long before the growth failure becomes clinically apparent [7,37,38]. In the current study, only 1.8% of children presented hypothyroidism. As there were no cases of a TSH level above 10 µIU/mL, all were subsequently classified as having subclinical hypothyroidism. The observed differences between the GHD and GHN group were not significant. 

The last aspect analyzed here is the vitamin D status. As vitamin D is one of the crucial regulators of calcium phosphate-metabolism, disorders of this metabolism may affect the process of growth [7]. Mutual relationships between the vitamin D and GH/IGF-1 axis have been observed, but they are complex and not clearly understood. GH and IGF-1 seem to be involved in vitamin D metabolism, increasing 1,25(OH)2-D3 production in kidneys; on the other hand, vitamin D seems to increase IGF-1 levels. Also, some research has shown lower vitamin D levels in GHD patients than in controls, with a prevalence of insufficiency and deficiency [3,39]. It has been postulated to evaluate vitamin D status in GHD- and rhGH-treated patients [3]. In our study group, among all 277 short stature children, we found the majority of them (72%) to have vitamin D levels below the optimal range, and only in 5.1% of all cases did the parents report supplementing their child’ diet with vitamin D regularly. There were no significant differences between the GHD and GHN group in terms of vitamin D deficiency, insufficiency or regular supplementation. This observation points to a substantial problem in basic pediatric care—it seems that vitamin D status should be taken into consideration in all children with growth retardation. 

The limitations of this study include primarily the relatively small sample size. For this reason, less pronounced differences may not have reached the level of statistical significance. However, we aimed to search for clear clinical differences, and these were not found. The collected data concerned only one medical center, which on the one hand resulted in a smaller size study group, but on the other hand meant uniform laboratory procedures, making the comparisons more reliable. Another limitation was that the study was retrospective in nature; therefore, it could only be based on observations and notes already made in the medical records. It was not possible to verify the data obtained, which in turn involves the risk of certain inaccuracies. Additionally, the study group was heterogeneous in terms of pubescence—both the GHN and GHD subgroups included prepubertal children as well as children and adolescents at various stages of sexual maturation. However, due to the nature of the study and varied descriptions of pubertal advancement in patient records, we were not able to perform an analysis in relation to the puberty stage. 

## 5. Conclusions

In view of all the above, the results obtained in our study point at major clinical similarities between the GHD and GHN subgroups. Therefore, distinguishing short-statured children with normal from those with deficient GH secretion, based on the cutoff level 10 ng/mL, seems rather artificial and not clinically evident. The only concrete difference was the result of peak GH concentration in stimulation tests on which the division into subgroups was made. On the one hand, these results suggest an overdiagnosis of GHD with the criteria used. Lowering the cutoff threshold would probably allow for a more reliable identification of children with GHD. However, on the other hand, the observed similarities also concern a clearly delayed bone age, a severity of height deficit, and IGF1 SDS values. It seems possible that among children with normal GH stimulation tests results, there were also those in whom endogenous, physiological secretion is insufficient, despite a proper reaction to pharmacological stimuli, and who could substantially benefit from rhGH treatment. As GHD diagnostic criteria appear not be fully reliable, maybe a wider availability for rhGH therapy based on the growth velocity and IGF-1 evaluation would be an option. We hypothesize that very short, slowly growing children, with GH peak levels in stimulation tests above 10 ng/mL, but decreased GH concentrations during physiological sleep tests, along with a delayed bone age and low IGF-1 SDS, would likely benefit from GH therapy. Nevertheless, the modifications of local diagnostic and therapeutic guidelines with different approaches to GH stimulation testing should be considered.

## Figures and Tables

**Table 1:** Characteristics of the study group—mean values and standard deviations (SD) for the analyzed variables in all short stature children, divided into subgroups depending on peak growth hormone (GH) level.

	All Children with Short Stature (*n* = 277)			Children with GH Peak <10 ng/mL (*n* = 138)			Children with GH Peak ≥10 ng/mL (*n* = 139)		
	All	Boys	Girls	All	Boys	Girls	All	Boys	Girls
Number	277	181	96	138	88	50	139	93	46
Gender [%]	-	65.34%	34.66%	-	63.8%	36.2%	-	66.9%	33.1%
Bone age delay [years]	−1.50 ± 0.93	−1.56 ± 0.95	−1.38 ± 0.87	−1.54 ± 0.89	−1.55 ± 0.85	−1.50 ± 0.96	−1.47 ± 0.96	−1.56 ± 1.03	−1.23 ± 0.76
BA/CA ± SD	0.81 ± 0.13	0.79 ± 0.14	0.83 ± 0.12	0.80 ± 0.14	0.78 ± 0.13	0.83 ± 0.11	0.82 ± 0.14	0.81 ± 0.14	0.83 ± 0.13
hSDS ± SD	−2.67 ± 0.65	−2.64 ± 0.63	−2.74 ± 0.68	−2.68 ± 0.61	−2.66 ± 0.58	−2.71 ± 0.69	−2.67 ± 0.68	−2.62 ± 0.69	−2.81 ± 0.69
IGF-1 ± SD [ng/mL]	157.75 ± 91.62	155.81 ± 91.79	161.48 ± 91.63	149.78 ± 93.02	141.13 ± 82.62	164.84 ± 108.04	165.83 ± 89.77	169.68 ± 98.13	157.58 ± 68.89
IGF-1 SDS ± SD	−0.61 ± 1.03	−0.78 ± 0.97	−0.29 ± 1.07	−0.67 ± 1.08	−0.87 ± 1.03	−0.34 ± 1.09	−0.55 ± 0.99	−0.70 ± 1.06	−0.24 ± 1.06
TSH ± SD [µIU/mL]	2.07 ±0.95	1.96 ± 0.89	2.29 ± 1.03	2.19 ± 1.03	2.07 ± 0.94	2.42 ± 1.17	1.96 ± 0.85	1.87 ± 0.84	2.14 ± 0.80
fT4 ± SD [ng/dL]	1.09 ± 0.19	1.08 ± 0.17	1.10 ± 0.24	1.08 ± 0.19	1.09 ± 0.20	1.06 ± 0.18	1.10 ± 0.20	1.07 ± 0.13	1.15 ± 0.30
25OHD ± SD [ng/mL]	25.36 ± 11.15	25.48 ± 11.25	25.81 ± 11.26	25.11 ± 10.05	24.54 ± 9.48	26.06 ± 11.10	25.62 ± 12.72	26.38 ± 12.67	25.00 ± 11.54
HGB ± SD [g/dL]	13.47 ± 7.97	13.80 ± 10.03	12.88 ± 0.93	12.92 ± 1.01	13.00 ± 1.00	12.74 ± 0.99	14.02 ± 11.26	14.57 ± 13.98	13.02 ± 0.82
Birth weight ± SD [g]	3065.8 ± 657.55	3119.2 ± 655.75	2967.3 ± 652.82	3118.0 ± 627.20	3148.2 ± 624.0	3064.8 ± 635.35	3015.1 ± 687.09	3093.8 ± 690.00	2861.3 ± 661.90

GH, growth hormone; BA/CA, bone age/calendar age index; hSDS, height SDS; IGF-1, insulin-like growth hormone 1; TSH, thyrotropin; FT4, thyroxine; 25OHD, 25-OH vitamin D; HGB, hemoglobin.

**Table 2:** Analysis of additional health conditions evaluated during the diagnostic process in children with short stature—together and divided into subgroups depending on peak growth hormone (GH) level.

	All Children with Short Stature (*n* = 277)			Children with GH Peak <10 ng/mL (*n* = 138)			Children with GH Peak ≥10 ng/mL (*n* = 139)		
	All	Boys	Girls	All	Boys	Girls	All	Boys	Girls
Number	277	181	96	138	88	50	139	93	46
Celiac disease	11 (4%)	6 (3.3%)	5 (5.2%)	6 (4.3%)	4 (4.5%)	2 (4%)	5 (3.6%)	2 (2.2%)	3 (6.5%)
Hypothyroidism *	5 (1.8%)	2 (1.1%)	3 (3.1%)	3 (2.2%)	0 (0.0%)	3 (6.0%)	2 (1.4%)	2 (2.2%)	0 (0.0%)
Anemia	8 (2.9%)	6 (3.3%)	2 (2.1%)	6 (4.3%)	4 (4.5%)	2 (4.0%)	2 (1.4%)	2 (2.2%)	0 (0.0%)
Vitamin D deficiency	100 (36.1%)	64 (35.4%)	36 (37.5%)	49 (35.5%)	34 (38.6%)	15 (30.0%)	51 (36.7%)	30 (32.3%)	21 (45.7%)
Vitamin D insufficiency	101 (36.5%)	67 (37.2%)	34 (35.4%)	58 (42.3%)	37 (42.5%)	21 (42.0%)	43 (30.9%)	30 (32.3%)	13 (28.3%)
Regular intake of vitamin D	14 (5.1%)	9 (5.0%)	5 (5.2%)	5 (3.6%)	2 (2.3%)	3 (6.0%)	9 (6.5%)	7 (7.5%)	2 (4.3%)
SGA	56 (20.2%)	34 (18.4%)	22 (22.9%)	25 (18.2%)	15 (17.2%)	10 (20%)	31 (22.3%)	19 (20.4%)	12 (26.1%)

Vitamin D deficiency: serum level below 20 ng/mL; vitamin D insufficiency: serum level between 20 and 30 ng/mL; SGA: small for gestational age; * subclinical hypothyroidism, TSH level did not exceed 10 µIU/mL in any case.

## Data Availability

Data analyzed during this study are available on a reasonable request.
